# PPARG expression in colorectal cancer and its association with staging and clinical evolution ^1^

**DOI:** 10.1590/s0102-865020200070000008

**Published:** 2020-08-17

**Authors:** Andre Luiz Prezotto Villa, Rogério Serafim Parra, Marley Ribeiro Feitosa, Hugo Parra de Camargo, Vanessa Foresto Machado, Daniela Pretti da Cunha Tirapelli, José Joaquim Ribeiro da Rocha, Omar Feres

**Affiliations:** I MSc, Division of Coloproctology, Department of Anatomy and Surgery , Medical School , Universidade de São Paulo (USP), Ribeirao Preto - SP , Brazil . Conception and design of the study; acquisition, analysis and interpretation of data, manuscript writing.; II PhD, Division of Coloproctology, Department of Anatomy and Surgery , Medical School , USP , Ribeirao Preto - SP , Brazil . Manuscript writing, critical revision.; III PhD, Department of Anatomy and Surgery , Medical School , USP , Ribeirao Preto - SP , Brazil . Analysis and interpretation of data, statistics analysis.; IV MD, Department of Anatomy and Surgery , Medical School , USP , Ribeirao Preto - SP , Brazil . Analysis of data.; V MD, Department of Anatomy and Surgery , Medical School , USP , Ribeirao Preto - SP , Brazil . Analysis and interpretation of data.; VI PhD, Department of Anatomy and Surgery , Medical School , USP , Ribeirao Preto - SP , Brazil . Analysis and interpretation of data, critical revision.; VII PhD, Associated Professor, Head, Division of Coloproctology, Department of Anatomy and Surgery , Medical School , USP , Ribeirao Preto - SP , Brazil . Critical revision, final approval.; VIII PhD, Associated Professor, Division of Coloproctology, Department of Anatomy and Surgery , Medical School , USP , Ribeirao Preto - SP , Brazil . Conception and design of the study, analysis and interpretation of data, critical revision, final approval.

**Keywords:** Colorectal Neoplasms, Biomarkers, Prognosis, Drug Therapy

## Abstract

**Purpose:**

To evaluate the gene expression of peroxisome proliferator activated receptors gamma (PPARG) in colorectal tumors and to correlate this data with clinical variables of the patients.

**Methods:**

We analyzed the gene expression of PPARG in 50 samples of colorectal tumors using real-time reverse transcription polymerase chain reaction, and 20 adjacent normal tissue samples as control. The results of these quantifications were correlated with the respective patients’ medical records’ clinical information.

**Results:**

PPARG expression was not different in the tumor tissue compared to the control tissue. Patients older than 60 years, histological type with mucinous differentiation, more advanced staging at the time of diagnosis, and patients who evolved with recurrence of the disease or death did not present higher PPARG expression.

**Conclusion:**

Expression of PPARGD was not associated with worse prognosis.

## Introduction

Colorectal cancer (CRC) represents one of the most common cancers in Brazil and in the world, with >200,000 new cases reported each year in United States ^1^ . Cancer is now widely recognized as a threat to global development. The rates of colorectal cancer are rising among adolescent and young adult patients-defined ^2 , 3^ . The increase of early-onset CRC incidence suggests more prevention initiatives are urgently warranted for young adults in the near future. Targeted and effective prevention measures are still needed among elderly populations ^4^ . In addition, recognition of prognostic factors and surgical treatment combined with chemotherapy has contributed to enhancing cure and survival rates in patients with colorectal cancer (CRC) ^5^ .

The most important prognostic factor in patients with these tumors is TNM staging. Notwithstanding the usefulness of this classification in selecting patients for specific treatment, TNM staging might not suffice as many patients with the same staging have distinct clinical outcomes. In this context, new biomarkers that can identify disease behavior on an individual basis are essential to improve patient care ^6 - 9^ .

PPARs (Peroxisome proliferator activated receptors) are nuclear receptors that antagonize transcription of immunity and inflammation factors. Monocytes, macrophages, T cells, dendritic cells, skeletal muscle cells, adipocytes, and gastrointestinal epithelial cells express PPARG (gamma), which is involved in lipid and glucose homeostasis and adipocyte inflammation and differentiation, among other activities ^10 - 12^ . PPARG is a member of the nuclear receptor superfamily of ligand activated transcription factors ^13^ . Ligand activation of PPARG induces it to heterodimerize with the retinoid X receptor and directly regulates gene expression. In addition, PPARG activation has shown to repress proinflammatory genes via transrepression and transcriptional squelching ^14^ .

PPARG is related to beta-catenin, an important molecule in colorectal tumor carcinogenesis ^15 - 18^ and studies have shown that PPARG plays an important role in regulating the growth of a number of different cancers, including colorectal cancer ^13 , 14 , 19^ .

PPARG immunohistochemical expression has been associated with good prognosis for CRC ^17^ . Some authors identified PPARG expression in 22% of CRC and correlated PPARG expression with better prognosis irrespective of other variables ^20^ . However, other studies have reported contrasting results and have shown that more PPARs may be linked to patient prognosis. PPARA (alpha) immunohistochemical expression in colorectal hepatic metastasis has been associated with worse overall survival as compared to cases that do not express this gene. This association has not been confirmed for PPARG ^21^ . PPARD (delta) promotes tumorigenesis and is probably clinically relevant: elevated PPARD and COX-2 expression in tumor tissues has been correlated with worse prognosis in patients with CRC ^22 , 23^ .

Literature studies clearly do not agree on the real role played by PPARs in carcinogenesis, but they provide evidence and theories that support both their anticancer and pro-carcinogenic activities ^24^ . Thus, the aim of this study was to correlate PPARs with colon rectal cancer prognosis and evolution.

## Methods

### *Study population and data collection*

The project was approved by the Research Ethics Committee of HCFMRP-USP, protocol number 1.469.953, on 30 ^th^ March 2016.

A retrospective cohort study involving 50 sequential patients with colon and rectum adenocarcinoma stages I to IV. These patients underwent surgical resection from 2008 to 2009 and were treated or not with adjuvant chemotherapy. The tumor specimens’ samples were stored in at -80 ^o^ C. The controls consisted of 20 normal, non-tumoral tissue samples that were adjacent to the neoplasm and which were collected during the same surgical procedure. The patients’ clinical, laboratory, and anatomopathological data were obtained during a search of medical charts.

### *Inclusion criteria*

We included patients with colon and rectal cancer with:

- Histological confirmation of colon or rectum adenocarcinoma;
- Availability of preserved tumor sample (stored under nitrogen) for RNA extraction and analysis;
- Patients exclusively treated at our institution.

### *Exclusion criteria*

We excluded patients with the following characteristics:

- Patients with inflammatory bowel disease;
- Patients with hereditary CRC;
- Tumors that were not adenocarcinoma; e.g., epidermoid carcinomas and intestinal stromal tumors.

### *PPARG RNA gene expression quantification*

The total RNA samples were extracted from tumoral and normal (adjacent to the tumor) tissues. The RNA integrity and concentration were quantified with the aid of a spectrophotometer. To synthesize cDNA (complementary DNA) from the mRNA, reverse transcription was conducted with the commercially available High Capacity cDNA Reverse Transcription Kit (Applied Biosystems); the manufacturer’s instructions were followed.

Amplification via real-time reverse transcription polymerase chain reaction (RT-PCR) was accomplished with TaqMan Master Mix (Applied Biosystems) by using the cDNA obtained from the tumor sample. To analyze gene expression quantitatively, the commercially available TaqMan Assay, consisting of oligonucleotides and probes (Applied Biosystems), was employed.

Given the differences caused by the distinct amounts of cDNA used in the reactions, the CT values determined for the different samples were normalized. The CT determined for a certain gene in a sample was subtracted from the CT determined for a housekeeping gene (GAPDH and IGHMBP2 in the present case) in the same sample, to give ∆CT. The ∆CT values could then be compared in different ways for the same gene, to give the gene relative quantification in distinct samples. The number of copies generated by a PCR reaction doubled after each cycle.

In this way, the number of cycles that separated the ∆CT of a sample from the ∆CT of a reference sample (∆∆CT) reflected the difference between the samples. The relative gene expression of the sample was approximately calculated by using the formula 2 ^-^ . All the reactions were performed in duplicate and analyzed on the 7500 Fast Real-Time PCR System (Applied Biosystems). The data were constantly collected during PCR and analyzed with the software Sequence Detection System, in order to obtain the CT values.

The reference genes (housekeeping genes) GAPDH and IGHMBP2 were selected on the basis of the literature and of new banks that list multiple data for a more adequate choice. GAPDH is traditionally used as reference gene in RT-PCR investigations concerning colon and PPARG. Studies continue to show that it is one of the most stable genes for control assays ^25 , 26^ . For this reason, GAPDH was chosen as one of the housekeeping genes.

The Genevestigator database (http://www.genevestigator.com) contains a large amount of information about different organisms and pathologies ^27^ . It offers search for reference genes with a more stable behavior and considers various biological contexts, such as the gene that will be compared and the species and pathology that will be investigated, among others ^28^ . With these data, the present experimental biological context was submitted to analysis by Genevestigator, which showed that IGHMBP2 was a stable gene for our research.

The current consensus is that the use of multiple genes to normalize the RT-PCR data is the best option ^29^ . For this reason, here both GAPDH and IGHMBP2 were used to evaluate PPARG expression on the basis of the average of the two genes.

### *Statistical analysis*

The Mann-Whitney test was applied to compare non-paired groups with non-Gaussian distribution. The Kruskal-Wallis test was employed if more than two groups had to be compared. The software Graphpad Prism 6.0 was used to carry out the statistical analyses and to construct the graphs. Significance was set at p < 0.05.

## Results

### *Patient’s sample analysis*

The clinical and pathological of the patients are illustrated in Table 1 . In summary, 50% of patients were male, 10% were < 45 years, 30% had 45-60 years, and 60% had > 60 years at diagnoses. The primary topography of cancer was rectum in 44% and 56% in the colon. The majority of cancer staging T3, without lymph nodes involvement (N0, 54%).

**Table 1:** Patient´s clinical and pathological characteristics.

Clinical and pathological characteristics	N (%)
Sex	
Male	25 (50)
Female	25 (50)
Age in years	
<45	5 (10)
45-60	15 (30)
>60-75	19 (38)
>75	11 (22)
Primary tumor topography	
Colon	28 (56)
Rectum	22 (44)
Staging pT	
T1	1 (2)
T2	5 (10)
T3	33 (66)
T4	11 (22)
Staging pN	
N0	27 (54)
N1	10 (20)
N2	13 (26)
Liver metastasis	
Presence	11 (22)
Absence	39 (78)
TNM	
I	1 (2)
II	21 (42)
III	17 (34)
IV	11 (22)
Angiolymphatic invasion	
Present	18 (36)
Absence	18 (36)
No information	14 (28)
CEA	
<5 ng/dL	27 (54)
5 to 15 ng/dL	10 (20)
>15 a 50 ng/dL	10 (20)
>50 ng/dL	3 (6)
Mucinous differentiation	
Presence	8 (16)
Absence	42 (84)

### *Staging and age and clinical evolution*

Only one patient, aged 58 years, had stage I tumor. As for stage II, III, and IV tumors, we did not notice any predominance of age range. However, the proportion of patients aged over 75 years increased progressively with more advanced tumor staging. Liver metastasis was present in 11 patients during staging. Patients of stage I, II and III had R0 resection. All rectal tumors were limited to the upper segment (intraperitoneal rectum), and none of these patients underwent neoadjuvant treatment. In all patients, it was performed an oncological resection with lymphadenectomy.

Regarding the postoperative treatment of the primary tumor lesion, 76% of the patients received chemotherapy, either as an adjuvant or palliative, reflecting the large percentage of patients who were in clinical stages III and IV, unequivocal indications of systemic therapy. Ten out of 21 patients of stage II and all patients of stage III and IV underwent chemotherapy. All patients underwent adjuvant chemotherapy with 5-fluorouracil and leucoverin.

**Figure 1:**
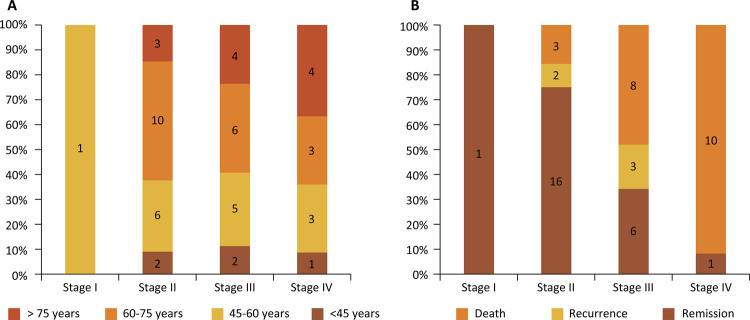
A. Correlation between age and tumor staging (Stage I to IV); B. Correlation between staging (Stage I to IV) with clinical evolution (death, recurrence or remission).

The mean follow-up of patients was at least 5 years. Although most of the patients in the study had more advanced stages of the disease, 68% of the patients were alive after more than 5 years of follow-up. Of these, 46% were disease-free and 12% had local and / or distant. Ten of the 11 patients stage IV died. The only surviving patient was submitted to resection of the hepatic metastasis and chemotherapy and was disease-free in July 2015. The patient that did not come for follow-up was considered as deceased on the basis of age (78 years) and presence of pulmonary lesions at the time of diagnosis. Of a total of 17 stage III patients, eight died, three presented recurrent disease, and six are in remission. All these patients received adjuvant chemotherapy. Of the 21 stage II patients, only three died, two had recurrent disease, and 16 are in remission. The one stage I patient has no evidence of disease (Fig. 1).

### *PPARG expression*

**Figure 2:**
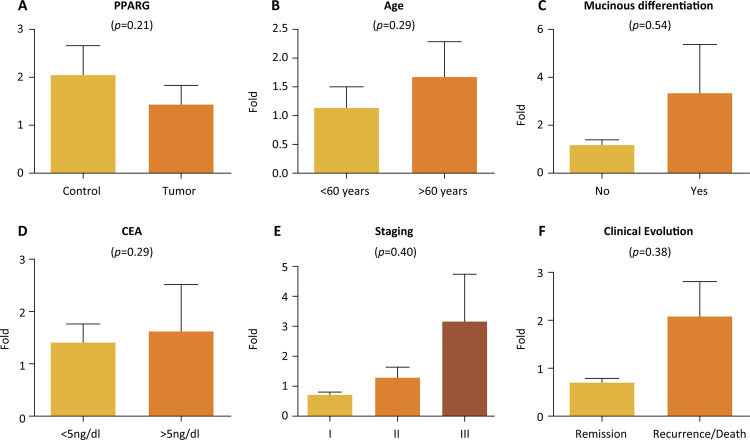
PPARG gene expression according to: A. tumor samples and samples of normal tissue adjacent to the tumor; B. Age; C. Histological type, regarding the presence or not of mucinous differentiation; D. CEA levels; E. Staging; F. Clinical evolution.

We compared the gene expression results of 49 tumor samples and 20 samples of normal tissue adjacent to the tumor, which were used as controls. One of the tumor samples did not present satisfactory gene amplification for RT-PCR, so we discarded it. We analyzed the data considering the reference genes GAPDH and IGHMBP2 as controls, and we used the average of both these genes. We observed that PPARG expression was compared to the control group (p = 0.2114). Twenty patients were aged 60 years or less, whereas 29 patients were older than 60 years. PPARG expression was compared in patients aged over 60 years (p = 0.2940), in patients with mucinous differentiation (p = 0.5452), in patients with CEA levels higher or lower than 5 ng/dL (p = 0.2949), in patients with more advanced tumor stages (p = 0.4062), and in patients with recurrence and death compared to patients in remission (p = 0.3895). There was only one stage I patient in the casuistic, so we did not include this patient in the statistical analysis (Fig. 2).

## Discussion

Our study showed that PPARG expression was not associated with worse prognosis, not even a better prognosis, as indicated in the literature, despite a tendency of higher levels in more advanced cancers and in patients with recurrence or deceased. Although studies have shown that PPARG is expressed in colon-derived tumors and normal colonic mucosa, the consequence of this on patient outcome is unclear ^30^ .

Most of the studies on the role of PPARG have shown it plays an anticancer role ^20 , 30^ . Ogino *et al* . ^20^ demonstrate, using 2 large, prospective, cohort-based studies, that the expression of PPARG in tumors is associated with increased survival compared with PPARG-negative tumors. Although this study does not address the role of PPARG in the development of colorectal cancer, it provides strong data that PPARG expression in tumors is associated with increased survival. This study represents a large study group from which strong, statistically based conclusions could be achieved, something lacking in the previous studies. Using univariate analysis or taking into account other potential indicators of patient outcome using multivariate analysis, patients with PPARG positive tumors showed a significant reduction in overall risk of mortality. Similar to smaller studies, just like ours, they investigated whether a number of clinical factors (age, gender, family history, year of diagnosis, tumor stage or grade) were playing a modifying effect on the association between PPARG expression and patient survival. These variables did not seem to modify the effect of PPARG on survival.

As previously mentioned, our study showed that PPARG expression was not associated with prognosis. Previous publications showed that PPARG is present at high levels in both normal and malignant colon tissues ^31 , 32^ . There are studies that evaluated the potential role of PPARG to function as tumor suppressor, a small clinical trial in advanced colorectal cancer was carried out using the Troglitazone, a potent activator of the PPARG ^33^ . However, no difference in disease progression or survival was observed and this activator of the PPARG was not an active agent for the treatment of metastatic colorectal cancer. Another study demonstrated that the use of PPARG ligands was associated with a decrease in lung, prostate, and colon cancer incidence ^34^ .

Some studies on CRC have pointed out that PPARG silencing is related to worse prognosis due to higher cancer cell proliferation and invasive potential ^17^ . In 2009, some authors analyzed PPARG expression by immunohistochemistry and found that only 22% of the tumors expressed PPARG, which translated into better clinical evolution ^20^ . These data conflict with our findings that PPARG RNA is expressed in almost all the studied cases even though quantification by RT-PCR shows higher expression in more advanced stages and in patients with worse prognosis. This difference could be explained as follows: whereas immunohistochemical expression requires sufficiently intact molecular and genetic machinery, RNA expression may be elevated without this machinery being in operation, probably due to mutations accumulated by the neoplasm. Hence, there might even be PPARG hyper-expression at the genetic level, but this higher expression does not translate into adequate receptor functioning or higher immunohistochemical expression.

In an attempt to explain why anticancer response is not always constant, consistent, and reproducible, some authors theorized that the cellular microenvironment controls PPARG activity from the outside to the inside of the cell ^35^ . In this sense, the receptor is only the intracellular part of a message that started being transmitted from the outside. Therefore, when a ‘disease threshold’ is reached, PPARG is epigenetically silenced: it no longer functions and is no longer expressed in the membranes, making the patient more susceptible to the disease. This may be the reason why some authors have described immunohistochemical expression of functioning PPARG as a factor for good prognosis. As for PPARG RNA expression, it might not translate into higher immunohistochemical expression in more advanced stages of the disease-after a certain stage of the disease, epigenetic silencing turns off PPARG despite its high gene expression.

This rationale is known as “nuclear receptor exhaustion theory”. In the initial stages, PPARG balances the detrimental effects of cancer and obesity and is fully functioning. In the event of disease progression, epigenetic mechanisms silence transcription factors, to reduce expression of membrane receptors. This is a plausible explanation for the high PPARG RNA expression in advanced stages of the disease, which could be a response of the organism to the poorly functioning or to the non-functioning PPARG in these stages. However, due to transcription silencing, this higher RNA expression does not reflect on viable and visible membrane receptors during immunohistochemistry.

Girgun G. made an editorial in the past describing that PPARG can be a new independent marker for colorectal cancer survival ^30^ . He describes that the studies suggest that PPARG ligands may not be effective as a single agent in advanced cancer, but may be able to prevent tumorigenesis. However, the author argues that the question remains as to the consequence of PPARG in colorectal tumors. Two previous studies investigated the role of PPARG expression on patient survival ^36 , 37^ . Similar to our results, neither of these studies found an association between PPARG expression and patient outcome. Perhaps in part due to the small sample size in that study and in ours. The author finishes his editorial explaining that, therefore, increased PPARG expression may be associated with decreased inflammation and would thus be predicted to result in a more indolent tumor. It would be of interest for future studies to determine if inflammatory markers were indeed reduced in the tumors expressing PPARG.

This study has some important limitations. First, literature data are still conflicting and inconclusive, but more detailed studies involving a higher number of patients from various institutions may help to infer the prognosis and therapeutic importance of this receptor, which could improve patient care. In addition, the immunohistochemistry analysis was not performed to prove that PPARG expression is not associated with prognosis. Finally, we included a small number of patients in our study. Maybe, if we had included a large number of patients, it would have impaired the statistics.

## Conclusion

PPARG expression was not associated with worse prognosis, and not even a better prognosis, as indicated in the literature, despite a tendency of higher levels in more advanced cancers and in patients with recurrence or deceased.
